# Impact of Hydrogel Spacer Insertion on Radiation Dose to Erectile Structures and Longitudinal Sexual Function in Prostate Cancer Patients

**DOI:** 10.3390/cancers18050814

**Published:** 2026-03-03

**Authors:** Eyael Zeru, Ziwei Feng, Liang Dong, Ning Meng, Yike Guo, Yi Luo, Yin Zhang, Holly Schuh, Kai Ding

**Affiliations:** 1 Department of Epidemiology, Johns Hopkins University, Baltimore, MD 21205, USA; hschuh1@jhu.edu; 2 Department of Electrical and Computer Engineering, Johns Hopkins University, Baltimore, MD 21218, USA; zfeng15@jhmi.edu; 3 Department of Urology, Ren Ji Hospital, Shanghai Jiao Tong University School of Medicine, Shanghai 200127, China; drdongliang@126.com; 4 Division of Rheumatology, Department of Medicine, Johns Hopkins University, Baltimore, MD 21287, USA; nmeng2@jhu.edu; 5 Department of Biomedical Engineering, Johns Hopkins University, Baltimore, MD 21287, USA; yguo122@jh.edu (Y.G.); yluo62@jh.edu (Y.L.); 6 Rutgers Cancer Institute of New Jersey, Rutgers, The State University of New Jersey, New Brunswick, NJ 08901, USA; yz718@cinj.rutgers.edu; 7 Department of Radiation Oncology and Molecular Radiation Sciences, Johns Hopkins University, Baltimore, MD 21287, USA

**Keywords:** prostate cancer, radiation therapy, erectile dysfunction, hydrogel spacer, sexual function, neurovascular bundle, penile bulb, dosimetry, radiotherapy toxicity, patient-reported outcomes

## Abstract

**Background:** Erectile dysfunction is a common late effect of prostate radiotherapy. Hydrogel spacers aim to reduce radiation exposure to nearby structures by increasing the distance between the prostate and surrounding tissues, potentially preserving sexual function. **Methods:** In this retrospective cohort study of 117 prostate cancer patients who received hydrogel spacers, we compared pre- and post-insertion radiation dose and anatomical positioning of erectile structures using paired t-tests. Longitudinal sexual function, assessed via EPIC scores, was modeled using linear mixed-effects regression with natural splines (df = 3), incorporating random intercepts and slopes to account for within-subject variability. **Results:** Spacer insertion significantly reduced radiation dose to the left and right neurovascular bundles (mean reductions: 1.66 Gy, 95% CI: 1.32–2.00; and 1.64 Gy, 95% CI: 1.28–2.01, respectively; *p* < 0.01) and the right perineal artery (1.33 Gy, 95% CI: 0.57–2.09; *p* < 0.01). No significant dose changes were observed for the penile bulb or left perineal artery, nor in anatomical distances. However, spatial displacement was confirmed by significant overlap and integrated volume changes. Longitudinal modeling showed a significant decline in sexual function between 12 and ≥36 months post-treatment (Spline 2: β = –12.72, 95% CI: −18.52–−6.92 and Spline 3: β = –6.68, 95% CI: −10.96–−2.40; *p* < 0.01). **Conclusions:** Hydrogel spacer insertion was associated with significant reductions in radiation dose to erectile structures, most notably the neurovascular bundles and the right perineal artery. However, longitudinal analyses revealed no corresponding preservation of sexual function. These findings suggest that while hydrogel spacers effectively reduce radiation exposure to key anatomical structures, their clinical benefit for maintaining erectile function remains uncertain.

## Simple Summary

Erectile dysfunction is a common long-term side effect for men receiving radiation therapy for prostate cancer. Hydrogel spacers are designed to protect nearby structures involved in sexual function by increasing the distance between the prostate and surrounding tissues. The aim of our retrospective study was to determine whether hydrogel spacer insertion reduces radiation exposure to erectile structures and whether this results in better long-term sexual function. In a cohort of 117 patients, we observed that hydrogel spacers lowered the radiation dose delivered to the neurovascular bundles and one of the perineal arteries and caused measurable displacement of surrounding anatomy. Despite these technical improvements, sexual function declined over time in a pattern consistent with typical post-radiation changes. These findings suggest that while hydrogel spacers effectively reduce radiation to key structures, this dose reduction alone may not be sufficient to preserve erectile function after treatment.

## 1. Introduction

Prostate cancer is one of the most commonly diagnosed malignancies among men, with an age-adjusted incidence rate of 114.7 cases per 100,000 individuals in the United States [1]. Radiation therapy (RT) is a primary treatment modality, offering effective tumor control while preserving organ function [2,3]. However, despite its therapeutic benefits, RT poses significant risks to surrounding tissues, including structures essential for erectile function [4]. The etiology of RI-ED is complex, as multiple anatomical structures, including the penile bulb, neurovascular bundles (NVBs), and pudendal arteries are at risk of radiation exposure [5,6]. While some studies suggest that higher radiation doses to the penile bulb are most strongly correlated with erectile dysfunction [7,8], others emphasize the role of neurovascular bundle and pudendal artery damage, leading to uncertainty about which structures are most critical for erectile preservation after RT [5,6,9,10,11,12].

To mitigate radiation-induced toxicity to surrounding organs, hydrogel spacers have been introduced in prostate radiotherapy [13,14]. These spacers increase the distance between the prostate and adjacent tissues, particularly the rectum, thereby reducing radiation exposure to critical structures [15,16,17,18]. While their effectiveness in lowering rectal toxicity is well-documented [19,20], their impact on erectile function preservation remains uncertain [21,22]. Some studies have reported that hydrogel spacers result in improved post-treatment sexual function, while others have found no significant benefit [22]. The mechanism by which hydrogel spacers influence erectile function remains unclear, and existing studies provide conflicting evidence regarding which anatomical structures are most affected by dose modifications [5,9,11]. Given these inconsistencies, further investigation is needed to determine whether hydrogel spacers reduce radiation exposure to erectile structures and whether these dosimetric changes translate into better long-term sexual function outcomes.

This study evaluated the dosimetric impact of hydrogel spacer insertion on radiation exposure to critical erectile structures and assessed longitudinal trends in sexual function following treatment, as measured by repeated EPIC sexual function scores. The Expanded Prostate Cancer Index Composite (EPIC) is a validated instrument designed to assess health-related quality of life across five key domains affected by prostate cancer treatment: urinary incontinence, urinary irritation, bowel, sexual, and hormonal function [23]. In this study, we focused on the sexual function domain of the EPIC score to evaluate patient outcomes following radiotherapy. EPIC is particularly useful for tracking recovery and comparing quality of life across treatment modalities over time [24,25,26,27]. Based on prior studies, EPIC sexual summary scores greater than 75 are generally associated with normal sexual function, scores between 61 and 75 reflect mild sexual dysfunction, and scores below 61 correspond to moderate to severe dysfunction [28]. By leveraging a dataset of prostate cancer patients treated with hydrogel spacers, this study compared actual radiation dose exposure to estimated doses that would have been received in the absence of a spacer, derived from CT-based modeling. This approach enabled a direct evaluation of how hydrogel spacer placement influenced radiation distribution to erectile structures. Additionally, longitudinal changes in erectile function were assessed using EPIC sexual function scores, modeled over time with a mixed-effects regression to understand overall trends following treatment.

## 2. Materials and Methods

### 2.1. Study Design and Data Source

This multi-institutional retrospective cohort study evaluated the impact of hydrogel spacer insertion on radiation dose to erectile structures and characterized longitudinal trends in post-treatment sexual function. Pre- and post-insertion dosimetric and spatial metrics were compared using paired *t*-tests, and EPIC sexual function scores were analyzed over time using a linear mixed-effects model.

The present study used data from a multi-institutional phase III hydrogel spacer trial (ClinicalTrials.gov identifier NCT01538628), which employed standardized contouring, treatment-planning, and follow-up protocols across participating centers. The analysis was based on secondary, de-identified patient data collected from 20 clinical institutions between 2012 and 2016. Patients received conventionally fractionated image-guided intensity-modulated radiotherapy (IG-IMRT) to a total dose of 79.2 Gy in 1.8-Gy fractions (44 fractions) to the prostate ± seminal vesicles, consistent with the phase III hydrogel spacer trial dataset used in the present analysis [13,29]. The dataset included radiotherapy treatment plans, contoured structures, dosimetric values, and patient-reported outcome scores measured using the sexual function subdomain of the Expanded Prostate Cancer Index Composite (EPIC).

Data was obtained from clinical imaging, radiation oncology planning systems, and patient surveys collected before and after treatment. The source database originated from a combination of institutional electronic medical records and radiation oncology systems.

### 2.2. Study Population

The initial cohort included 222 participants, but after applying inclusion and exclusion criteria, 117 participants were included in the final analysis. The cohort comprised predominantly low-grade disease: 77 patients (65.8%) had ISUP grade 1 tumors, 34 (29.1%) grade 2, and 6 (5.1%) grade 3; no patients had ISUP grades 4–5. Patients in the dataset underwent external beam radiation therapy (EBRT) for prostate cancer with hydrogel spacer placement prior to treatment.

#### 2.2.1. Inclusion Criteria

- Histologically confirmed diagnosis of prostate cancer.
- Clinical stage 1 or 2 disease, based on the TNM (Tumor, Node, Metastasis) classification system.
- Treated with external beam radiation therapy (EBRT) and hydrogel spacer placement.
- Availability of radiation therapy planning data both with and without hydrogel spacers, including CT-based estimated pre-spacer plans and actual post-spacer plans.
- Availability of dosimetric or spatial data for at least one of the erectile structures: Penile bulb (PB), Neurovascular bundles (NVBs), Perineal arteries (PNAs).
- Availability of at least two non-missing EPIC sexual function scores across baseline and follow-up time points.

#### 2.2.2. Exclusion Criteria

- Complete absence of all pre- and post-spacer dosimetric and spatial data for the penile bulb, NVBs, and PNAs.
- Fewer than two recorded EPIC sexual function scores over the study period.
- Subjects who are indicated for androgen deprivation therapy or who have been treated with androgen therapy within the prior 3 months.

### 2.3. Variables and Measurements

The primary exposures of interest were radiation doses delivered to erectile-related anatomical structures, specifically the penile bulb (PB), neurovascular bundles (NVBs), and perineal arteries (PNAs). Dosimetric and anatomical variables were extracted from pre- and post-hydrogel radiotherapy treatment plans and included:

- Penile Bulb (PB): Volume, overlap volume, integrated volume, mean radiation dose (Gy), and minimum distances to prostate clinical landmarks (prostate contour line [CL], clinical target volume [CTV], and planning target volume [PTV]).
- Neurovascular Bundles (NVBs): Left and right NVB volumes, combined overlap volume, and mean radiation doses (Gy).
- Perineal Arteries (PNAs): Left and right PNA volumes, combined overlap volume, and mean radiation doses (Gy).

Overlap volume was used to assess spatial displacement, defined as the volume of tissue remaining in the same location before and after hydrogel spacer insertion—lower overlap values implied greater displacement. For the NVBs and PNAs, overlap volume was calculated as a combined value across both sides. Integrated volume reflected the overall spatial extent of pre- and post-insertion anatomy, with higher values indicating more displacement.

Patient-reported outcomes were measured using the Expanded Prostate Cancer Index Composite (EPIC) sexual function domain scores, collected at baseline and at 3, 6, 12, 15, and ≥36 months post-treatment. Higher scores reflect better sexual function. Only subjects with at least two non-missing EPIC sexual scores were included to ensure sufficient data for longitudinal analysis.

### 2.4. Study Outcomes

The primary outcome was the change in sexual function over time following hydrogel spacer insertion, assessed using longitudinal EPIC sexual function scores. While descriptive statistics summarize average sexual scores at each time point, these cross-sectional averages do not fully account for within-subject variability. Therefore, to account for within-subject variability and the correlated nature of repeated measures, we applied a linear mixed-effects model incorporating natural splines. This approach allowed for a nuanced analysis of nonlinear trends in sexual function over time.

The secondary outcome was the evaluation of spatial and dosimetric changes following hydrogel insertion. Specifically, we compared pre- and post-insertion radiation doses to the PB, NVBs, and PNAs, and assessed anatomical displacement through overlap and integrated volumes.

### 2.5. Data Processing and Statistical Analysis

All statistical analyses were conducted using R (version 4.4.3). Descriptive statistics, including means and standard deviations, were calculated for key anatomical and dosimetric variables before and after hydrogel spacer insertion. These variables included the volume, radiation dose, and spatial displacement metrics (overlap and integrated volumes) for the penile bulb (PB), neurovascular bundles (NVBs), and perineal arteries (PNAs).

To evaluate the effect of hydrogel insertion on these parameters, paired *t*-tests were used to compare pre- and post-insertion values for each structure. This allowed for direct assessment of spatial and dosimetric changes within the same individuals.

Longitudinal trends in sexual function were analyzed using a linear mixed-effects model. The EPIC sexual function domain scores, collected at multiple follow-up time points, served as the outcome variable. Time was modeled as a continuous variable using natural cubic splines with three degrees of freedom to flexibly capture non-linear patterns in sexual function recovery or decline. To account for individual variability, the model included both random intercepts and random slopes for time at the subject level, reflecting differences in baseline function and post-treatment trajectories. All statistical analyses were conducted using R (version 4.4.3; accessed on 7 April 2025). The model was implemented using the lme4 and lmerTest packages in R.

All statistical tests were two-sided, and a *p*-value less than 0.05 was considered statistically significant.

### 2.6. Ethical Considerations

This retrospective observational study analyzes secondary, de-identified patient data to evaluate the dosimetric effects of hydrogel spacer use in prostate cancer radiotherapy and its association with erectile function. The dataset was originally collected from 20 institutions and contains no identifiable patient information.

The study was reviewed by the Johns Hopkins Bloomberg School of Public Health Institutional Review Board (IRB) and was determined not to constitute human subjects research. Therefore, it did not require IRB oversight (IRB Determination Notice dated 12 December 2024). All data were handled in compliance with institutional and ethical standards for research involving de-identified health information.

## 3. Results

Table 1 presents the mean sexual function scores and standard deviations at each follow-up time point after hydrogel spacer insertion. These descriptive statistics reflect the available sample at each time point and do not account for repeated measures or intra-individual changes over time.

A noticeable decline in mean EPIC scores was observed between baseline (Mean = 53.3, SD = 24.4) and 3 months post-treatment (Mean = 45.8, SD = 24.1), with modest variation across subsequent follow-up periods. While scores slightly increased at 6 and 15 months, they remained consistently below baseline throughout the 36-month period.

Table 2 summarizes spatial changes and dosimetric outcomes before and after hydrogel spacer insertion. The average minimum distances between the PB and the prostate clinical target volumes (CTV, PTV, and Prostate CL) remained relatively stable following hydrogel insertion, with slight increases observed in PB-to-CTV and PB-to-PTV distances. Mean radiation dose to the PB decreased marginally post-insertion, from 19.43 Gy to 18.76 Gy. Similarly, the mean dose to both the left and right NVBs declined modestly. In contrast, mean doses to the left and right PNAs remained largely unchanged.

To assess spatial displacement of these structures following hydrogel insertion, overlap and integrated volumes were analyzed. The overlap volume reflects the portion of a structure occupying the same spatial position before and after hydrogel placement, with lower values suggesting greater displacement. The integrated volume represents the combined spatial extent of the structure pre- and post-insertion, with higher values implying more movement overall.

On average, PB volume increased slightly from 3.58 cm^3^ to 3.74 cm^3^. The PB overlap volume was 1.93 cm^3^ (SD 1.11), and the integrated volume was 5.10 cm^3^ (SD 1.73), indicating moderate positional change. NVB volume remained stable (3.50 cm^3^ pre- and post-insertion), though the low overlap volume of 1.66 cm^3^ (SD 1.10) suggests a measurable shift in location. The PNA volume showed a slight decrease post-insertion (from 4.23 cm^3^ to 4.09 cm^3^), with an overlap volume of 2.44 cm^3^ (SD 1.23), also pointing to spatial displacement.

**Figure 1:**
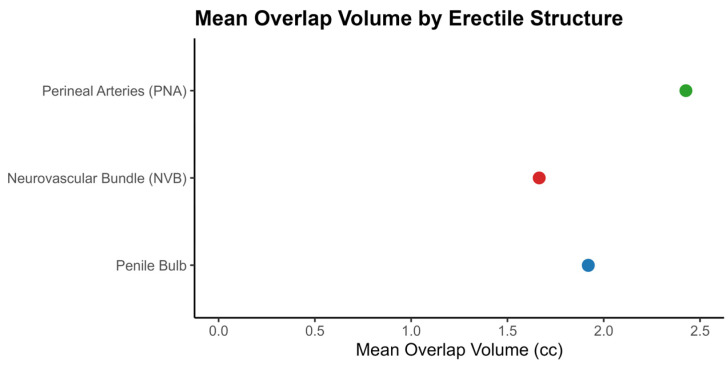
Mean Post-Treatment Overlap Volume of Erectile Structures Exposed to Radiation.

We present the mean post-treatment overlap volumes to illustrate the magnitude of anatomical displacement experienced by erectile structures following hydrogel spacer insertion in Figure 1.

**Figure 2:**
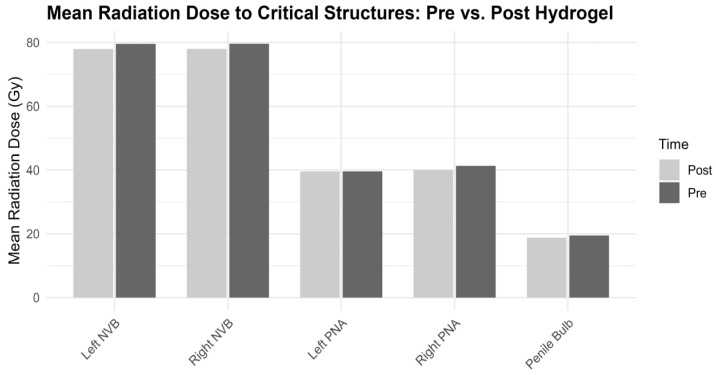
Mean Radiation Dose to Critical Erectile Structures Before and After Hydrogel Spacer Insertion.

We also display the mean radiation dose to these critical erectile structures before and after spacer placement in Figure 2.

A series of paired *t*-tests were conducted to assess whether hydrogel spacer insertion significantly altered the anatomical positioning and radiation exposure of critical structures associated with erectile function, namely, the penile bulb (PB), neurovascular bundles (NVBs), and perineal arteries (PNAs).

No statistically significant changes were observed in the mean distances between the PB and the prostate, clinical target volume (CTV), or planning target volume (PTV) following hydrogel insertion (all *p* > 0.05). Similarly, there was no significant change in the mean dose to the penile bulb (pre: 19.43 Gy, post: 18.76 Gy; *p* = 0.16) or to the left perineal artery (pre: 39.5 Gy, post: 39.5 Gy; *p* = 0.91).

However, statistically significant dose reductions were observed in several structures:

**Left NVB**:

- Mean dose decreased from 79.5 Gy to 77.9 Gy (*p* < 0.01; 95% CI: 1.32 to 2.00)
- Maximum dose decreased from 83.4 Gy to 83.1 Gy (*p* < 0.01; 95% CI: 0.22 to 0.39)

**Right NVB**:

- Mean dose decreased from 79.6 Gy to 77.9 Gy (*p* < 0.01; 95% CI: 1.28 to 2.01)
- Maximum dose decreased from 83.4 Gy to 83.2 Gy (*p* < 0.01; 95% CI: 0.10 to 0.34)

**Right PNA**:

- Mean dose decreased from 41.3 Gy to 39.9 Gy (*p* < 0.01; 95% CI: 0.57 to 2.09)

These findings support a dose-sparing effect of hydrogel spacer insertion, particularly for the neurovascular bundles and the right perineal artery.

Additionally, combined overlap and integrated volumes, representing total anatomical displacement rather than side-specific values, were all statistically significant.

- PB Overlap Volume: 1.93 cm^3^ (95% CI: 1.62 to 2.24; *p* < 0.0001)
- PB Integrated Volume: 5.10 cm^3^ (95% CI: 4.61 to 5.59; *p* < 0.0001)
- NVB Overlap Volume: 1.66 cm^3^ (95% CI: 1.30 to 2.01; *p* < 0.0001)
- PNA Overlap Volume: 2.44 cm^3^ (95% CI: 2.05 to 2.82; *p* < 0.0001)

These significant values indicate measurable anatomical displacement following hydrogel insertion, although they reflect total rather than side-specific overlap, an important consideration when interpreting spatial-dose relationships.

Table 3 summarizes the dosimetric and spatial displacement metrics before and after hydrogel spacer insertion for erectile function–related structures.

### 3.1. Mixed Effects Model Analysis of Sexual Function Scores

We analyzed longitudinal changes in EPIC sexual function scores using a linear mixed-effects model with natural cubic splines (df = 3) to flexibly capture nonlinear trends over time. The final model included random intercepts and random slopes for time splines to account for both individual differences in baseline sexual function and variation in change trajectories across patients.

#### 3.1.1. Model Selection and Fit Comparison

To guide model selection, we first evaluated whether including radiation dose variables improved model fit over a base model that included only time. As shown in Table 4, the likelihood ratio test comparing the full model (with neurovascular bundles, perineal artery, and penile bulb variable doses) to the base model (time only) revealed no significant improvement in model fit (*p* = 0.1774). Additionally, the inclusion of covariates reduced the analytic sample from 117 to 102 subjects, largely due to missing data. Consequently, we retained the base model to maximize sample size and statistical power.

Next, we compared the base model with time modeled using 2 versus 3 degrees of freedom. The model with df = 3 provided a significantly better fit (AIC = 5207.9 vs. 5216.2; *p* = 0.0026, Table 5), indicating that additional flexibility better captured the nonlinear trend in sexual function over time.

#### 3.1.2. Final Model Output

Table 6 presents fixed and random effect estimates from the final model. The trajectory of sexual function scores showed a significant decline at later follow-up periods. Specifically, the second spline component (Estimate = –12.72, *p* < 0.001) and third component (Estimate = –6.68, *p* = 0.003) were significantly negative, indicating sharper declines during mid- to long-term follow-up. The first spline component (Estimate = –1.71) was not statistically significant (*p* = 0.55), suggesting no abrupt change immediately post-treatment.

The model accounted for substantial variability across individuals. The variance in random intercepts (σ^2^ = 461.0; SD = 21.47) reflects wide variation in baseline sexual function between patients. Similarly, the random slopes for the spline terms showed high variability (σ^2^ = 453.7 [SD = 21.30] for spline 1 and σ^2^ = 422.7 [SD = 20.56] for spline 2), indicating that individual patients experienced different patterns of change in sexual function over time. The residual variance was 103.8 (SD = 10.19), representing moderate within-person variability across follow-up points.

Table 7 summarizes the random-effect variance and standard deviation estimates from the final mixed-effects model.

**Figure 3:**
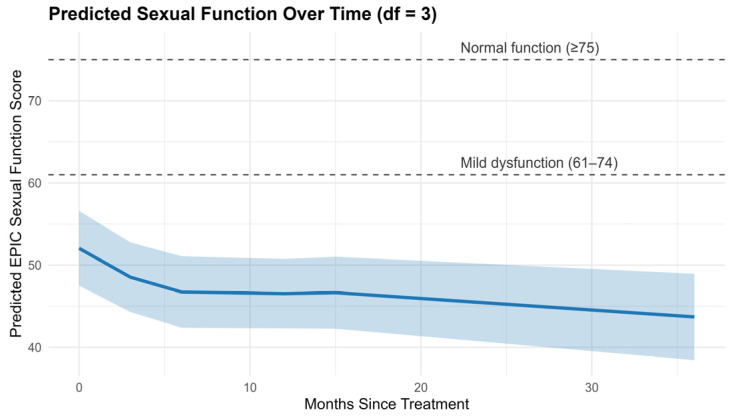
Predicted Sexual Function Trajectory Over Time Based on Mixed Effects Model.

A plot of the predicted trajectory from the final model (Figure 3) illustrates a clear decline in sexual function following radiotherapy, with the steepest drop observed between 12 and 36 months post-treatment.

## 4. Discussion

This study evaluated the impact of hydrogel spacer insertion on radiation exposure to erectile-related structures and analyzed longitudinal trends in sexual function among prostate cancer patients. Spacer insertion was associated with modest but statistically significant reductions in radiation dose to the neurovascular bundles (NVBs) and right pudendal artery, as well as measurable anatomical displacement of surrounding structures, indicated by significantly non-zero overlap and integrated volume metrics. While descriptive statistics of EPIC sexual scores showed persistently low mean values at each follow-up time point, these cross-sectional averages reflect different subsets of patients at each interval and do not account for within-subject trajectories. Therefore, we relied on a linear mixed-effects model to more accurately characterize longitudinal changes in sexual function. Despite the dosimetric and anatomical changes observed, the model revealed a consistent decline in sexual function over time, with no evidence of functional preservation attributable to spacer use.

The reductions in radiation dose to the NVBs and right perineal artery reaffirm the spacer’s intended effect [6,19,30], creating physical separation between the prostate and critical structures to reduce radiation exposure [31]. These benefits were further supported by spatial displacement data: overlap volumes (the volume shared between pre- and post-insertion locations) and integrated volumes (the total spatial extent across time) both indicated that hydrogel placement caused meaningful shifts in the position of the erectile structures. While these values were statistically significant, it is important to note that the overlap volumes for the NVBs and PNAs were calculated as combined metrics across both sides, limiting their interpretability in side-specific analyses.

Despite these improvements, no corresponding enhancement in sexual function was observed. Longitudinal modeling using a linear mixed effects framework, with random intercepts and slopes and natural splines to flexibly capture time effects, demonstrated a sustained decline in EPIC sexual scores over time. This decline was especially prominent in the mid and later phases of follow-up. No meaningful recovery was observed over time. This pattern is consistent with the known time course of radiation-induced erectile dysfunction, which tends to worsen gradually after treatment [32]. The variability in baseline sexual function (σ^2^ = 461.0, SD = 21.47) and trajectory over time (spline variances ranging from σ^2^ = 215.4 to 453.7) underscores the heterogeneity in patient experience, highlighting that individual factors likely play a significant role in functional outcomes.

Importantly, a likelihood ratio test comparing a time-only model to models incorporating post-insertion radiation dose metrics revealed no improvement in model fit (*p* = 0.177). Including these covariates also reduced the sample size due to missing data, which further supported the decision to retain the time-only model. This allowed us to preserve the full cohort of 117 patients and maximize statistical power. While this model could not directly evaluate the relationship between radiation dose and sexual outcomes, the persistent decline in scores suggests that functional deterioration continues even when dose to erectile structures is reduced.

Several limitations warrant consideration. First, this was a retrospective analysis, and unmeasured confounding (e.g., baseline erectile function, age, comorbidities, use of androgen deprivation therapy) could influence both dosimetric exposure and functional outcomes. Second, we lacked a non-spacer control group, limiting causal inference about the spacer’s clinical impact. Third, and importantly, we did not have side-specific overlap volumes for the NVBs or PNAs, which restricted our ability to perform regression analyses linking spatial displacement to dose for each structure independently. While we reported the combined displacement metrics and found them statistically significant, their lack of anatomical specificity limits conclusions about directional dose-sparing effects. The study also did not evaluate directional components of hydrogel-induced displacement (e.g., anterior–posterior vs. cranio-caudal), which may further clarify structure-specific dose sparing and warrants investigation in future studies.

Although hydrogel spacer insertion effectively reduces radiation dose to key erectile structures, these technical improvements did not correspond with functional preservation in our cohort. This finding underscores a critical clinical insight: dosimetric gains alone may not be sufficient to prevent long-term erectile dysfunction. Future prospective studies with larger sample sizes are needed to enhance generalizability and statistical power. In addition, incorporating comparator arms, comprehensive patient-level variables, and side-specific anatomical data will be important to better define which patients benefit most from hydrogel spacer placement.

## 5. Conclusions

Hydrogel spacer insertion resulted in measurable anatomical displacement and meaningful reductions in radiation dose to several erectile structures, particularly the neurovascular bundles and the right perineal artery. These findings confirm the dosimetric benefits of spacer placement in prostate radiotherapy and support its role in reducing exposure to tissues that contribute to erectile function.

Despite these technical improvements, long-term sexual function declined across all follow-up periods, with no evidence that spacer use preserved erectile outcomes. The lack of functional improvement highlights the multifactorial nature of radiation-induced erectile dysfunction and suggests that dose reduction alone may be insufficient to maintain sexual health. Future studies incorporating comprehensive patient-level factors, prospective designs, and comparison groups are needed to clarify which patients may benefit most from hydrogel spacer placement and to determine whether additional strategies are required to effectively protect sexual function.

## Figures and Tables

**Table 1:** Mean EPIC Sexual Function Scores Following Hydrogel Spacer Insertion.

Visit Time Point	Mean Sexual Score	Standard Deviation (SD)	Upper Bound (Mean + SD)	Lower Bound (Mean − SD)
Baseline	53.3104	24.3901	77.7005	28.92037
3 months	45.7992	24.0629	69.86212	21.73624
6 months	49.6182	26.7158	76.33401	22.90246
12 months	46.2889	25.4137	71.70256	20.87524
15 months	47.6285	25.1993	72.82786	22.42918
≥36 months	45.4331	23.4612	68.89429	21.97182

**Table 2:** Spatial Changes and Dosimetric Outcomes Pre- and Post-Hydrogel Insertion.

Structure/Variable	Pre-Mean (SD)	Post-Mean (SD)
Penile Bulb Volume (cm^3^)	3.58 (1.64)	3.74 (1.61)
PB Overlap Volume (cm^3^)	-	1.93 (1.11)
PB Integrated Volume (cm^3^)	-	5.10 (1.73)
NVB Volume (cm^3^)	3.50 (1.55)	3.50 (1.72)
NVB Overlap Volume (cm^3^)	-	1.66 (1.10)
PNA Volume (cm^3^)	4.23 (1.87)	4.09 (1.72)
PNA Overlap Volume (cm^3^)	-	2.44 (1.23)
Minimum Distance (PB to Prostate CL, mm)	15.73 (4.22)	15.71 (4.30)
Minimum Distance (PB to CTV, mm)	15.00 (5.21)	15.77 (5.08)
Minimum Distance (PB to PTV, mm)	8.82 (4.98)	9.50 (5.44)
Mean Dose to Penile Bulb (Gy)	19.43 (15.45)	18.76 (17.01)
Mean Dose to Left NVB (Gy)	79.52 (2.71)	77.86 (4.42)
Mean Dose to Right NVB (Gy)	79.56 (2.67)	77.92 (4.57)
Maximum Dose to Left NVB (Gy)	83.4 (1.24)	83.1 (1.16)
Maximum Dose to Right NVB (Gy)	83.4 (1.32)	83.2 (1.48)
Mean Dose to Left Perineal Artery (Gy)	39.5 (7.77)	39.5 (8.88)
Mean Dose to Right Perineal Artery (Gy)	41.3 (9.28)	39.9 (9.66)
Left Perineal Artery Maximum Dose (Gy)	55.3 (11.52)	55.6 (13.61)
Right Perineal Artery Maximum Dose (Gy)	56.8 (12.03)	56.2 (12.82)

**Table 3:** Comparison of Dosimetric and Spatial Displacement Metrics Pre- and Post-Hydrogel Insertion for Structures Associated with Erectile Function.

Variable	Mean (Pre)	Mean (Post)	Mean Diff	95% CI (Diff)	*p*-Value *
Distance PB–Prostate (mm)	15.73	15.71	0.02	[−0.71, 0.76]	0.9505
Distance PB–CTV (mm)	15.00	15.77	−0.77	[−1.84, 0.31]	0.1600
Distance PB–PTV (mm)	8.82	9.50	−0.68	[−1.70, 0.34]	0.1906
Mean Dose to Penile Bulb (Gy)	19.43	18.76	0.73	[−0.29, 1.75]	0.1610
Mean Dose to Left NVB (Gy)	79.52	77.86	1.66	[1.32, 2.00]	<0.0001 ***
Mean Dose to Right NVB (Gy)	79.56	77.92	1.64	[1.28, 2.01]	<0.0001 ***
Left NVB Maximum Dose	83.4	83.1	0.31	[0.22,0.39]	<0.0001 ***
Right NVB Maximum Dose	83.4	83.2	0.22	[0.1,0.34]	0.0003 ***
Mean Dose to Left Perineal Artery (Gy)	39.5	39.5	0.03	[−0.58, 0.51]	0.9090
Mean Dose to Right Perineal Artery (Gy)	41.3	39.9	1.33	[0.57, 2.09]	0.0007 ***
Left Perineal Artery Maximum Dose	55.3	55.6	0.39	[−1.17, 0.40]	0.3350
Right Perineal Artery Maximum Dose	56.8	56.2	0.65	[−0.21, 1.50]	0.1380
Volume PB (cm^3^)	3.58	3.74	−0.16	[−0.66, 0.34]	0.5286
Overlap Volume PB (cm^3^)	-	1.93	-	[1.62, 2.24]	<0.0001 ***
Integrated Volume PB (cm^3^)	-	5.10	-	[4.61, 5.59]	<0.0001 ***
Volume NVB (cm^3^)	3.50	3.48	0.02	[−0.24, 0.28]	0.8971
Overlap Volume NVB (cm^3^)	-	1.66	-	[1.30, 2.01]	<0.0001 ***
Volume PNA (cm^3^)	4.23	4.13	0.10	[−0.18, 0.38]	0.4903
Overlap Volume PNA (cm^3^)	-	2.44	-	[2.05, 2.82]	<0.0001 ***

* Statistical significance levels: *p* < 0.05 (*), *p* < 0.001 (***).

**Table 4:** Comparison of Base vs. Full Model (df = 3).

Model	LogLik	df	χ^2^	*p*-Value *
Base (Time only)	−2245.9	15	-	-
Full (Time + Doses)	−2242.1	20	7.64	0.1774

* Statistical significance levels: *p* < 0.05 (*).

**Table 5:** Comparison of Base Model with df = 2 vs. df = 3.

Model	LogLik	df	χ^2^	*p*-Value *
df = 2	−2598.1	10	-	-
df = 3	−2598.0	15	18.28	0.0026 **

* Statistical significance levels: *p* < 0.05 (*), *p* < 0.01 (**).

**Table 6:** Final Base Model Estimates: Longitudinal Changes in EPIC Sexual Function Score (Natural Splines, df = 3).

Fixed Effects	Estimate	Std. Error	95% CI (Lower, Upper)	df	t Value	*p*-Value *
Intercept	52.00	2.20	[47.67–56.33]	116.36	23.67	<0.001 **
Spline 1 (Early trend)	−1.71	2.81	[−7.24–3.83]	114.16	−0.61	0.546
Spline 2 (Mid-phase drop)	−12.72	2.95	[−18.52–−6.92]	98.19	−4.32	<0.001 **
Spline (Late-phase drop)	−6.68	2.17	[−10.96–−2.40]	68.54	−3.08	0.003 **

* Statistical significance levels: *p* < 0.05 (*), *p* < 0.01 (**).

**Table 7:** Random Effects Summary.

Random Effect Term	Variance	Std. Dev.
Subject-level Intercept	461.0	21.47
Spline 1 Random Slope	453.7	21.30
Spline 2 Random Slope	422.7	20.56
Spline 3 Random Slope	215.4	14.68
Residual (within-subject variability)	103.8	10.19

## Data Availability

Due to privacy and institutional restrictions, these data are not publicly available. Aggregated data supporting the findings can be made available from the corresponding author upon reasonable request and with appropriate institutional approvals.

## Abbreviations

The following abbreviations are used in this manuscript:

RT	Radiation Therapy
RI-ED	Radiation-Induced Erectile Dysfunction
NVB(s)	Neurovascular Bundle(s)
PNA(s)	Perineal artery (or arteries)
PB	Penile Bulb
EPIC	Expanded Prostate Cancer Index Composite
CL	Contour line (used in “Prostate CL”)
CTV	Clinical Target Volume
PTV	Planning Target Volume
EBRT	External Beam Radiation Therapy
TNM	Tumor, Node, Metastasis
ADT	Androgen Deprivation Therapy
IRB	Institutional Review Board
